# Genomic Characterization of 30 Lytic *Klebsiella pneumoniae* Bacteriophages

**DOI:** 10.3390/v18101079

**Published:** 2026-09-30

**Authors:** Daniel A. Mora, Trisha Gryder, Lauren L. Lessor, Jordyn Michalik-Provasek, Michael J. Satlin, Thomas J. Walsh, Jason J. Gill

**Affiliations:** 1 Interdisciplinary Graduate Program in Genetics and Genomics, Texas A&M University, College Station, TX 77843, USA; danielmora@tamu.edu; 2 Center for Phage Technology, Texas A&M University, College Station, TX 77843, USA; 3 Department of Biochemistry & Biophysics, Texas A&M University, College Station, TX 77843, USA; 4 Department of Microbial Pathogenesis and Immunology, Texas A&M College of Medicine, College Station, TX 77843, USA; 5 Transplantation-Oncology Infectious Diseases Program, Department of Medicine, Weill Cornell Medicine, Cornell University, New York, NY 10065, USA; 6 Departments of Pediatrics and Microbiology & Immunology, Weill Cornell Medicine, Cornell University, New York, NY 10065, USA; 7 Center for Innovative Therapeutics and Diagnostics, Richmond, VA 23220, USA; 8 Department of Animal Science, Texas A&M University, College Station, TX 77843, USA

**Keywords:** *Klebsiella pneumoniae*, bacteriophage, characterization, genomics

## Abstract

The emergence and spread of Gram-negative pathogens that are resistant to multiple antibiotic classes pose a global risk to public health. Among such pathogens, carbapenem-resistant strains of *Klebsiella pneumoniae* (CR-KP) are of concern due to their resistance to treatment by most antibiotics and the mobility of their drug resistance determinants. Bacteriophages that target *K. pneumoniae* are a promising alternative antimicrobial option. Here, we characterize 30 lytic bacteriophages from various environmental sources using whole-genome sequencing, bioinformatic analysis, virulence assays, and assessment of the effects of media on phage sensitivity. Bioinformatic analysis revealed a diverse collection of phages that span 9 ICTV *Caudoviricetes* families and 13 genera with genome sizes ranging from 39 kbp to 349 kbp, representing all three tailed phage morphotypes. The phages were all found to be strictly lytic and able to inhibit bacterial growth, and no virulence or antibiotic resistance genes were detected within the phage genomes. Host range testing showed that the culture medium used for plating can have a significant effect on observed susceptibility of some isolates to phages, and phages infecting multiple strains showed greater variation between media. Analysis of phage tail fiber proteins indicated that phages in the collection are predicted to encode from one to nine receptor-binding proteins, with 17/30 phages encoding at least one tail spike with predicted capsular depolymerase activity. The lack of detectable depolymerase domains in 13/30 phages suggests a large portion of the phage collection may recognize non-capsule receptors. This study describes a collection of candidate phages for further development as potential antimicrobial agents against CR-KP and the complexity in understanding phage–host dynamics of phages that recognize non-capsule receptors in *K. pneumoniae*.

## 1. Introduction

*K. pneumoniae* is a Gram-negative, non-motile, rod-shaped bacterium in the *Enterobacteriaceae* family. It is known for its polysaccharide capsule, antibiotic resistance, and ability to be both a human commensal and opportunistic pathogen [1,2]. *K. pneumoniae* can colonize human mucosal surfaces and skin asymptomatically and is capable of causing a range of serious infections including pneumonia, liver abscesses, urinary tract infections, and sepsis [1]. *K. pneumoniae* can be acquired from healthcare or community settings, and infections have become difficult to treat due to its resistance to frontline antibiotics [1,3]. *K. pneumoniae* is part of a group of human pathogens commonly associated with high level of antibiotic resistance designated the ESKAPE pathogens that includes *Enterococcus faecium*, *Staphylococcus aureus*, *Klebsiella pneumoniae*, *Acinetobacter baumannii*, *Pseudomonas aeruginosa*, and *Enterobacter* spp. [4]. Reflecting the gravity of this global threat to public health, the World Health Organization listed carbapenem-resistant *K. pneumoniae* (CR-KP) as its top-ranked bacterium within the Bacterial Priority Pathogens List 2024 [2].

The rise of antibiotic resistance in *K. pneumoniae* is a threat to public health globally [2,4]. In 2022, it was estimated that 4.71 million deaths were associated with bacterial antimicrobial resistance, including 1.14 million deaths that were attributed to bacterial drug resistance [5]. The WHO has recognized carbapenem-resistant *K. pneumoniae* (CR-KP) as a pathogen that poses an urgent threat to public health [2]. The classification of CR-KP as a microorganism of concern is due to the high trend of antibiotic resistance, low preventability, and high mortality outcomes due to limited treatment options [2,6,7]. This is due to the fact that CR-KP harbor carbapenemase enzymes, which make them resistant to nearly all beta-lactam antibiotics, including carbapenems [8,9,10]. The most notable enzymes are the *bla*KPC alleles, which were first identified in the United States in 1996 and can hydrolyze a wide range of beta-lactams [11,12]. CR-KP has been implicated as the driver for the rising increase in carbapenem resistance globally [13,14]. The spread of drug resistance has led to an urgent need for antimicrobials effective against drug resistant pathogens [2,5].

A promising alternative to traditional antibiotics is phage therapy, which involves the use of phages as antimicrobial agents against pathogenic bacteria. Phages were first discovered in the early 20th century by Felix d’Herelle, who isolated an agent effective against Shiga dysentery [15]. d’Herelle coined the term bacteriophage (“bacteria-eater”) and pioneered the early use of phage therapy [15,16]. Phage therapy was largely abandoned in the 1940s following the introduction of antibiotics [16,17]. The rise of antibiotic resistance and challenges developing novel and effective antibiotics has prompted renewed interest in phage therapy [18,19,20].

There are several factors that make phages an attractive alternative antimicrobial agent. Phages are ubiquitous in nature, estimated at 10^31^ phage particles in the biosphere, and they can be readily isolated from the environment [21,22,23]. Additionally, phages are highly specific to their bacterial hosts and can typically only infect certain strains of their host species [24]. This specificity is largely due to the tail fibers or tail spikes of phages only recognizing specific receptors on the host cell surface [25]. This host specificity means that phages can target pathogens without disturbing the other members of the human microbiome [26]. These properties and demonstrated efficacy in reducing the bacterial burden in vitro and in vivo have renewed interest in the application of phage therapy [27,28,29,30,31,32].

There are over 1600 phages able to infect *Klebsiella* hosts listed in the NCBI virus database [33]. Phages that target *K. pneumoniae* have been isolated from a wide range of sources, including soil, water, sewage, and human feces [24,34]. Several studies have revealed a large genomic diversity of phages that can target *Klebsiella* spp. [24,34]. For the successful application of phage therapy, careful characterization of environmental phages is necessary. Phages must be analyzed to ensure they follow a strictly lytic lifecycle and do not carry any virulence or antibiotic resistance determinants [35]. In addition, efficacy in reducing bacterial load in vitro against clinically relevant strains is commonly assessed to identify suitable therapeutic phage candidates.

To address the urgent need for new antimicrobials against CR-KP, this work focuses on the isolation and characterization of 30 lytic phages that infect clinically relevant *Klebsiella* spp. strains. These phages were isolated from diverse environmental sources, including influent wastewater, activated sludge, surface waters, and swine feces. The phages include a variety of morphologies spanning nine ICTV families and 13 distinct genera [36,37]. We also measured each phage’s infectivity on different clinically isolated *Klebsiella* spp. and under various culture conditions. Together, the genomic and experimental data reveals each phage’s taxonomic identity and its potential for future use. The goal of this study is to present a comprehensive overview of these 30 *Klebsiella* spp. phages to lay the groundwork for selecting the most promising candidates as antibacterial agents against carbapenem-resistant *Klebsiella* spp.

## 2. Materials and Methods

### 2.1. Bacterial Strains and Culture Conditions

The *Klebsiella* strains used in this work are listed in Table 1. All culturing methods were carried out in either Luria Broth (LB) (10 g/L tryptone (BD, Franklin Lakes, NJ, USA), 10 g/L NaCl, and 5 g/L yeast extract (BD)), or tryptic soy broth (TSB) (Bacto TSB, BD), with aeration at 37 °C. Solid media (LB agar or tryptic soy agar (TSA)) were composed of the broth media amended with 15 g/L Bacto agar (BD). The *Klebsiella* clinical isolates were provided by Karen Frank of the NIH Clinical Center (NIH CC) or by Dr. Michael Satlin of Weill Cornell Medical College (WCMC) as denoted in Table 1. Antibiotic sensitivities of WCMC isolates were determined as described in [38].

### 2.2. Isolation and Culture of Bacteriophages

Phages were isolated over a seven year period (2012–2019). Phages were isolated by either direct plating filter-sterilized (0.22 µm, PES membrane, Millipore, Burlington, MA, USA) wastewater samples to host lawns or using a culture enrichment method, with 1–10 mL of filter-sterilized sample added to actively growing host cultures in TSB and incubated overnight at 37 °C with aeration [39]. Each phage was plaque purified three times to ensure clonality. Phages were routinely cultured and enumerated using the soft agar overlay method with TSA bottom plates overlaid with lawns of 5 mL top agar (15 g/L Bacto TSB, 4 g/L Bacto agar) inoculated with 100 µL of fresh overnight host culture and incubated overnight at 37 °C [40]. Phage plaque morphology was assessed by observing and photographing phage plaques as they appeared on soft overlay lawns. High-titer stocks were prepared by inoculating log-phase cultures with phage at a multiplicity of infection (MOI) of ~0.1 and incubating with aeration at 37 °C. Phage lysates were then centrifuged (10,000× *g*, 4 °C, 10 min) and filter sterilized by passage through 0.2 µm PES filters (Millipore, Burlington, MA, USA). Phages were routinely diluted in phage buffer (5.8 g/L NaCl, 1.0 g/L MgSO_4_·7H_2_O, and 25 mM Tris-HCl pH 7.4).

### 2.3. DNA Extraction and Sequencing

Phage genomic DNA was purified from lysates using a modified protocol from the Promega Wizard DNA purification kit as described previously (Promega, Madison, WI, USA) [41,42]. Bacterial DNA was prepared from fresh overnight cultures using the EZDNA Bacterial DNA kit (Omega Bio-Tek, Norcross, GA, USA) kit following manufacturer instructions. Phage genomic DNA was sequenced using 454 pyrosequencing (phages Poteet and Shiner) or Illumina (all others) at commercial sequencing providers. Bacterial DNA was sequenced by Illumina at a commercial provider.

### 2.4. Bioinformatics

Phage genomes sequenced using 454 sequencing were assembled with Newbler v2.7. Phages sequenced using Illumina were quality controlled with FASTQC and assembled using SPAdes v3.5.0 [43,44]. Phage genomes were closed using resolution of circular assemblies or PCR followed by Sanger sequencing. Genomes were annotated using the Center for Phage Technology’s Galaxy–Apollo phage genome annotation platform [45]. Bacterial genomes were assembled using SPAdes v3.5.0 and annotated using Prokka v1.14.5 [43,46]. Kaptive Web was used to determine the capsule (K) and LPS (O) antigen locus types of the 9 *Klebsiella* isolates used as phage hosts [47,48,49]. PathogenWatch [50] was used to determine the clonal groups, and Kleborate [51] was used to determine virulence and antimicrobial resistance determinants. CARD [52] was used to determine antibiotic resistance determinants. VFDB [53] was used to determine virulence determinants, and PHASTEST [54] was used to determine lysogeny determinants.

### 2.5. Host Range Testing

Phage host range was determined with spot testing using serially diluted phage lysates standardized to an initial titer of 10^8^ PFU/mL. Each standardized phage lysate was used to generate tenfold serial dilutions, and a 10 µL spot of these dilutions was plated onto a bacterial lawn, which was prepared as described above [55]. Phage plaques were enumerated, and the apparent titer was divided by the titer observed on the phage propagation host to calculate phage efficiency of plating (EOP) for each test strain.

### 2.6. Virulence Assay

Phage virulence was characterized against the propagation host using a microtiter plate-based liquid virulence assay [56]. The bacterial inoculum was prepared by subculturing bacterial overnight cultures in 1:100 in TSB and incubating with aeration at 37 °C to an OD_550nm_ of 0.25 (~10^8^ CFU/mL). Phage lysates were titered and adjusted to a concentration of 1 × 10^8^ PFU/mL. For each assay, 180 µL of adjusted bacterial inoculum in TSB was mixed with 20 µL of phage in sterile, untreated Falcon (Corning, Corning, NY, USA) 96-well transparent plates to achieve an initial MOI of 0.1. The plates were incubated at 37 °C with double orbital shaking in a Tecan Spark 10 M plate reader (Tecan Group Ltd., Männedorf, Switzerland), and growth was monitored by measuring OD_550nm_ at 15 min intervals for 20 h. All assays were performed in triplicate biological replicates. Resulting growth curves were visualized in GraphPad Prism 10 after adjusting readings against the baseline medium-only control [57].

## 3. Results

### 3.1. Characteristics of Carbapenem-Resistant Klebsiella spp. Clinical Isolates

A total of nine isolates of *Klebsiella* spp. were used in this study, as shown in Table 1 and Table 2. Multilocus sequence typing (MLST) analysis revealed that the nine isolates were distributed across eight different sequence types (Table 2) [50]. Two strains were identified as ST258, which has been implicated in the global spread of *K. pneumoniae* carbapenemases [58] and hospital outbreaks [59]. The remaining isolates were classified into individual STs: ST17, ST3585, ST3586, ST3861, ST359, ST35, and ST1502. The capsule (K) and lipopolysaccharide O antigen (O) loci were predicted bioinformatically using Kaptive, as shown in Table 1 [48]. All isolates had unique predicted K loci, whereas only three distinct O loci were identified. The predominant O locus type is O1/O2, with two predicted to have subtype O1/O2v1 and two noted as subtype O1/O2v2 [51]. Four isolates were predicted to have O5 locus type, and two isolates were predicted to have the O3 locus type. The remaining isolates were predicted to have O3b or O5 [51].

All *Klebsiella* spp. isolates provided by WCMC (Table 1) were bloodstream isolates collected in 2013 from New York or New Jersey medical centers as described in [38]. All but one of these isolates was found to encode a *bla*KPC beta-lactamase (Table 2). All were found to be resistant to cefotaxime, ceftazidime, cefepime, aztreonam, ertapenem, imipenem, and meropenem but were sensitive to ceftazidime–avibactam (see Supplementary Table S1).

Since the bacterial capsule has been shown to be a limiting factor for *Klebsiella* phages [24], each isolate was analyzed to predict its capsule type. The core genes that comprise the capsule of *Klebsiella* (K) locus are *galF*, *cpsACP*, *wzi*, *wza*, *wzb*, *wzc*, *wcaj/wbaP*, *gnd*, and *ugd*, which encode the core capsule biosynthesis machinery [60,61]. Analysis using Kaptive confirmed that eight of the nine strains contain the core capsule locus [48]. A single isolate, *K. pneumoniae* strain 39428, was bioinformatically predicted to have a nonfunctional capsule locus due to the truncation of a *wcaJ* gene.

### 3.2. Isolation, Genomic Characterization, and Diversity of Phages

**Figure 1:**
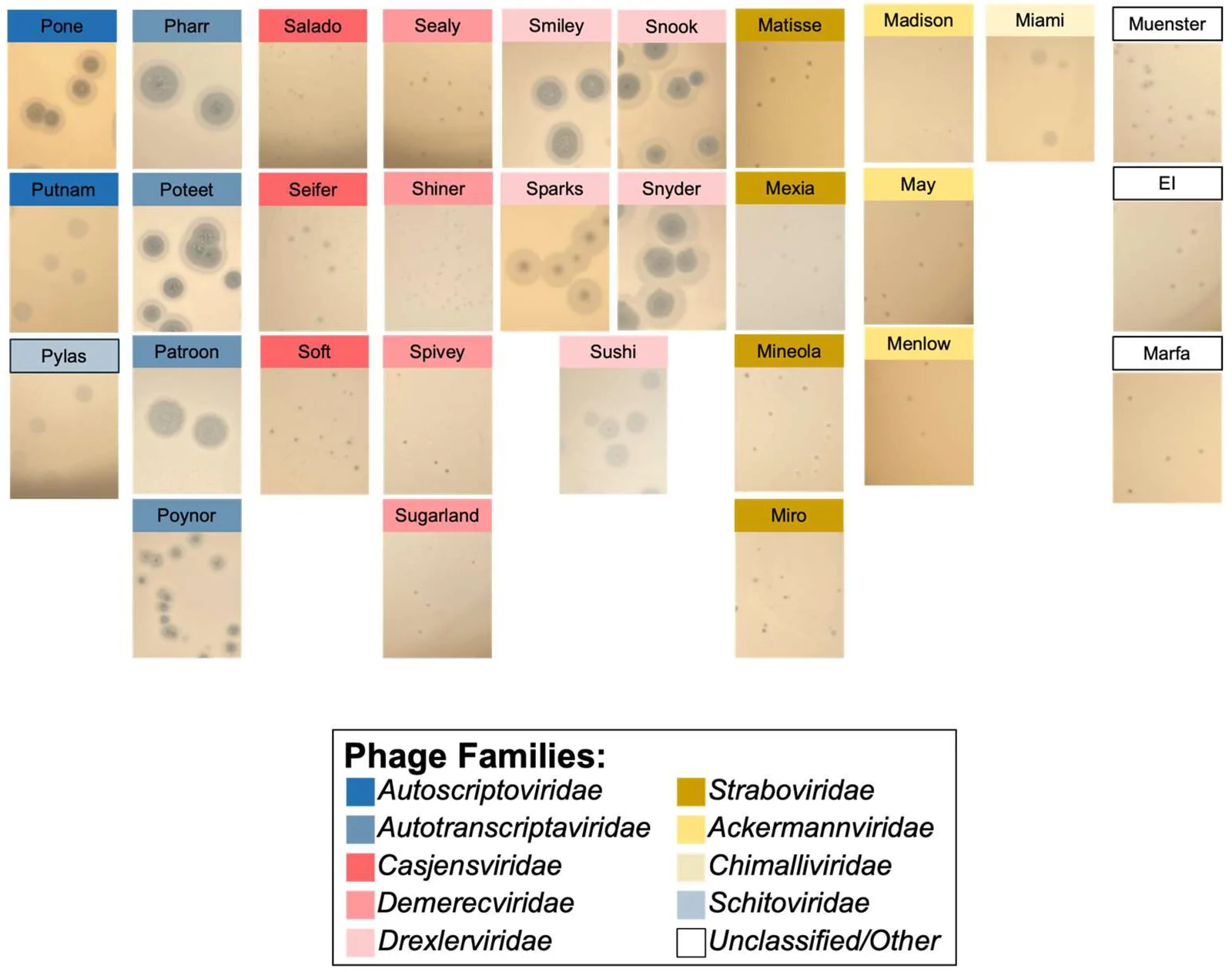
Plaque morphology of phage isolates. Phage names are colored based on ICTV taxonomic family. All *Autoscriptoviridae*, *Autotranscriptaviridae*, *Casjensviridae*, *Drexlerviridae*, *Schitoviridae*, *Chimallivirida*, and Muenster produce haloed plaques, consistent with the predicted presence of a capsular depolymerase tail spike. Phages Patroon and Poynor lack predicted enzymatic tail spikes, but their plaques are still surrounded by halo-like zones. *Casjensviridae* group phages produce small haloes and also are predicted to encode depolymerase-like tail spikes. This indicates that haloed plaque formation is not a reliable indicator of phage capsular depolymerase presence.

The 30 isolated phages capable of infecting the nine clinically isolated *Klebsiella* spp. (Table 3) were obtained from influent wastewater, activated sludge, surface waters, and swine feces around TX and isolated using standard isolation techniques [39]. These phages were generally named with the first letter associated with the morphotype (“M” for myophages, “S” for siphophages, and “P” for podophages) and named based on selected towns in Texas; the only exception to this system was the myophage EI. Three of the phages in this collection, Pharr, Soft, and Spivey, have been shown to have in vivo efficacy in treating mouse models of *Klebsiella* bacteremia and gastrointestinal translocation [27,31]. Examination of plaque morphology revealed that of the 30 phages isolated, fifteen phages formed diffuse halos (Figure 1).

Phage genomic DNA was purified from high titer stocks and sequenced, resulting in 30 novel, nonredundant phages cultured on nine *Klebsiella* spp. isolates (Table 3). The resulting genomes ranged in size from 39,442 bp (Patroon) to 346,937 bp (Muenster) in length with G+C contents between 31.86% and 56.45%. Phage morphotype was predicted based on phage phylogenetic relationships and the presence of genes encoding tail components.

The phages were classified into a family and genus based on the current ICTV taxonomy (2024) release. Of the 30 phages, 15 were already placed into the ICTV taxonomy (2024). To identify an ICTV taxa for the remaining phages, the unclassified phages were analyzed using BLASTn [76] to identify the closes relative with an ICTV taxonomic designation, which was used to determine phage family and genus (Table 3) [77]. The unclassified phages that showed less than 95% but more than 70% genome similarity were vB_KleM-RaK2 (Muenster), *Klebsiella* phage vB_KpnP_SU552A (Putnam), *Klebsiella* phage KPN N98 (Salado), *Klebsiella* phage ABTNL-2 (Snyder), and *Klebsiella* phage vB_KpnS-VAC5 (Sparks). The remaining phages displayed over 95% but less than 100% genome similarity: *Klebsiella* phage Menlow (Madison), *Klebsiella* phage Pharr (Poteet), *Klebsiella* phage 6996 (Poynor), *Klebsiella* phage vB_KpnS_Uniso31 (Sealy), *Klebsiella* phage vB_Kpn_B01 (Shiner), *Klebsiella* phage GZ9 (Smiley), *Klebsiella* phage vB_KpnD_PeteCarol (Snook), *Klebsiella* phage vB_KaeM_Nispero (Mexia), *Klebsiella* phage KP15 (Miro), and *Klebsiella* phage Marfa (EI).

**Figure 2:**
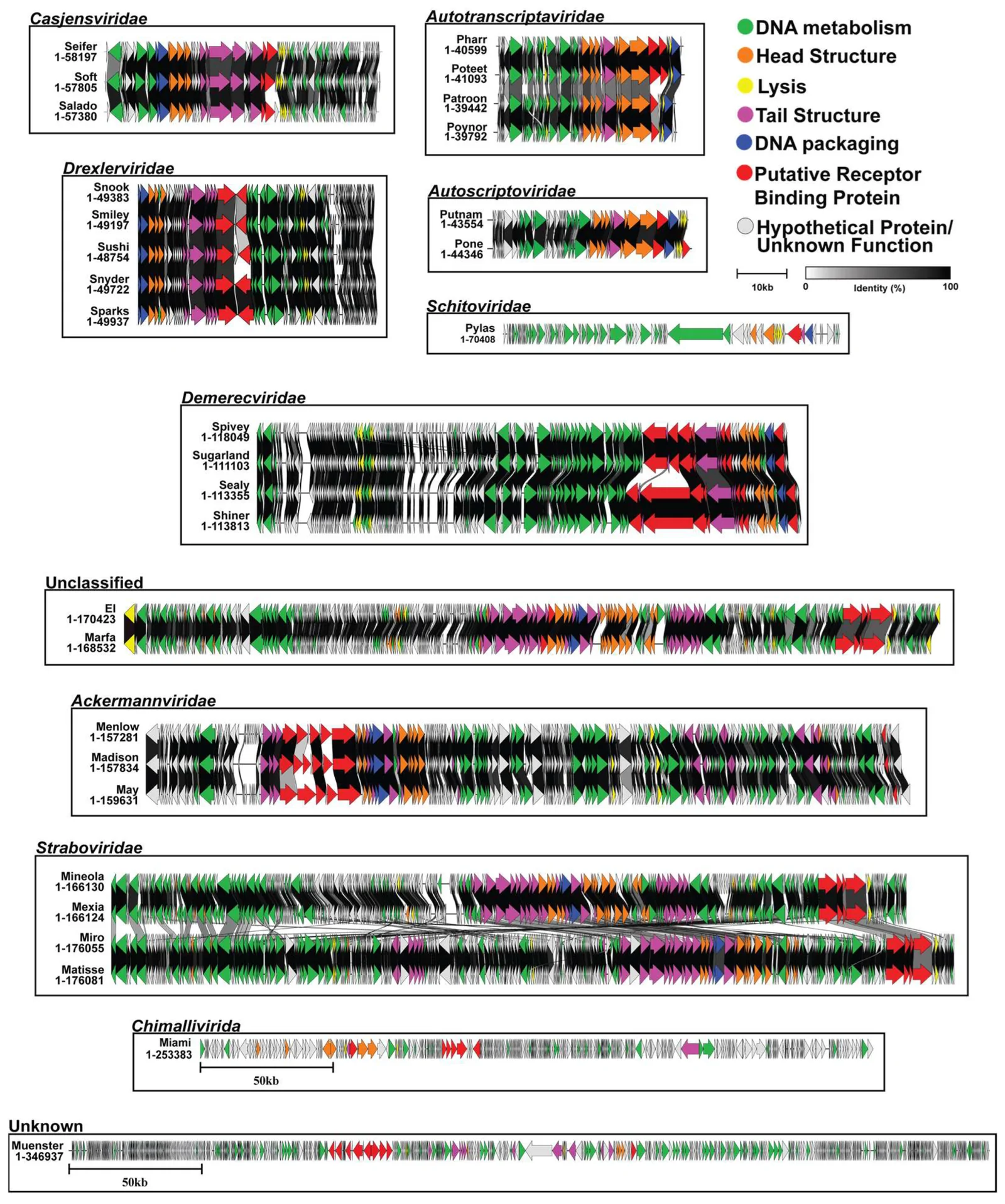
Genome similarity of isolated *Klebsiella* phages. The ICTV classification system was used to group and classify phages into the closest viral family and genus (if applicable). The genome organization of each phage is shown. The arrows represent protein-coding sequences and are color-coded based on functional categories. C-linker was used to perform global protein alignments and calculate similarities between clusters. Phages were named using the first letter to associate to the morphological group of the phage (M, myophage; P, podophage; S, siphophage).

**Figure 3:**
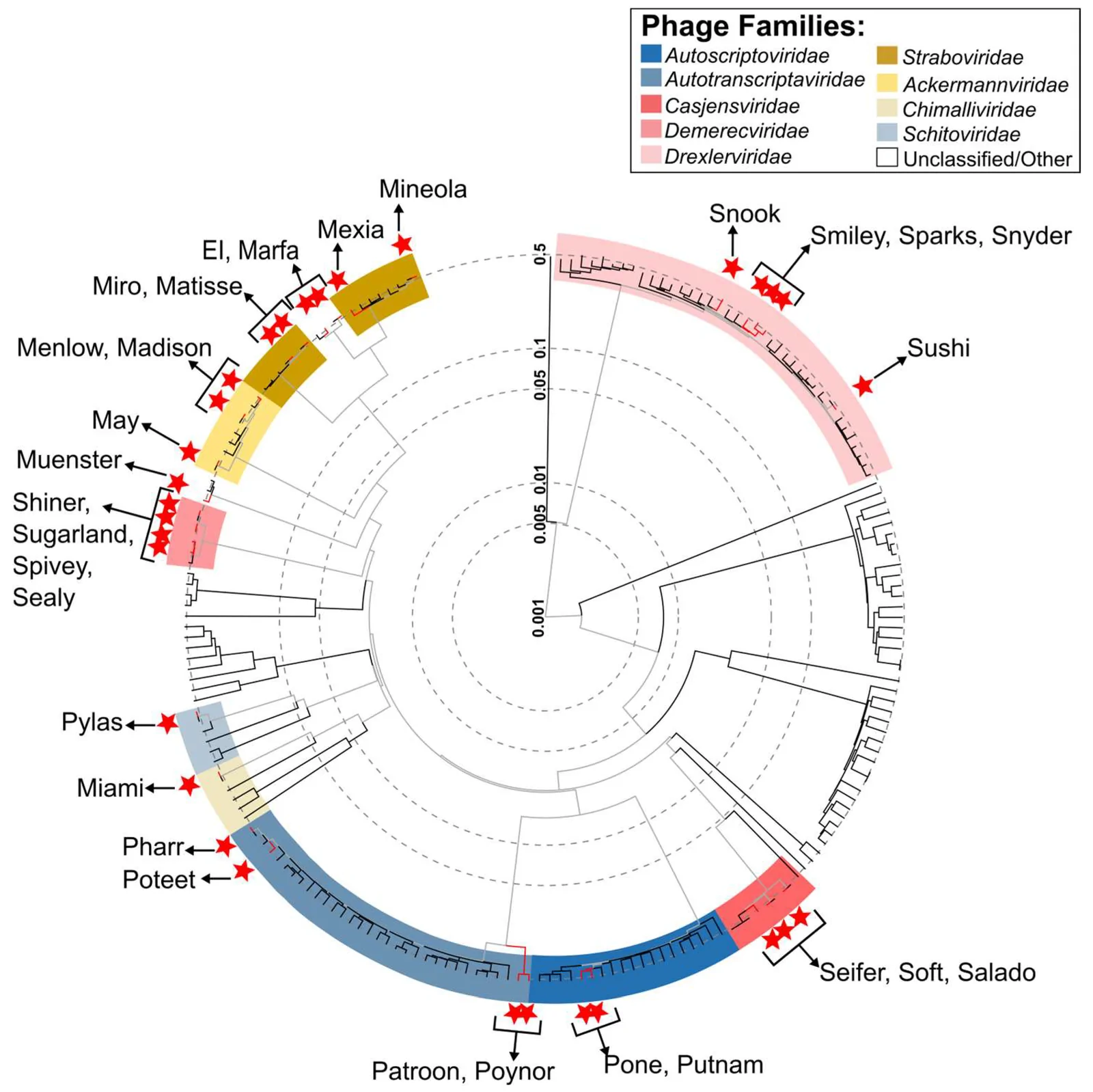
ViPTree protein-level phylogenetic tree of 30 *K. pneumoniae* phages. The proteomic phylogenetic tree was used to group phages based on genome wide sequence similarities. The analysis was restricted to include double-stranded DNA reference phages that infect Gram-negative bacteria of the phylum *Pseudomonadota*. This resulted in comparing 2404 of the 15,971 to the reference phages available on ViPTree against the phages in the collection to observe genome-wide proteomic relationships. Phage names are colored based on ICTV family designation.

The genomes of the phages encode between 47 and 567 genes. No virulence determinants, antimicrobial determinants, and known lysogeny genes were found in any phage genomes, indicating that the collection is composed entirely of virulent phages [52,53,54]. Functional genes were divided into seven groups based on putative function: DNA metabolism, head structure, lysis, tail fiber/tail structure, DNA packaging, and hypothetical function (Figure 2). A proteomic tree was constructed using ViPTree to show global relationships of the phages (Figure 3) [78]. This analysis showed the phages in the collection represented highly diverse phage types and supported the DNA-based taxonomic placement of the phages. Phages EI and Marfa are in an unclassified genus. However, based on the proteomic tree, they cluster most closely to other members of the *Straboviridae*; thus it would be appropriate to assign them to this family. Phage Muenster clusters with a deeply branching lineage that is not closely related to any other family and thus is likely a member of a novel taxonomic family. The proteomic tree was able to classify Muenster with *Klebsiella* phage vB_KleM_RaK2 and *Klebsiella* phage K64-1, which were also classified in ICTV under the *Alcyoneusvirus* genus.

### 3.3. Lytic Potential and Host Range of Phages

**Figure 4:**
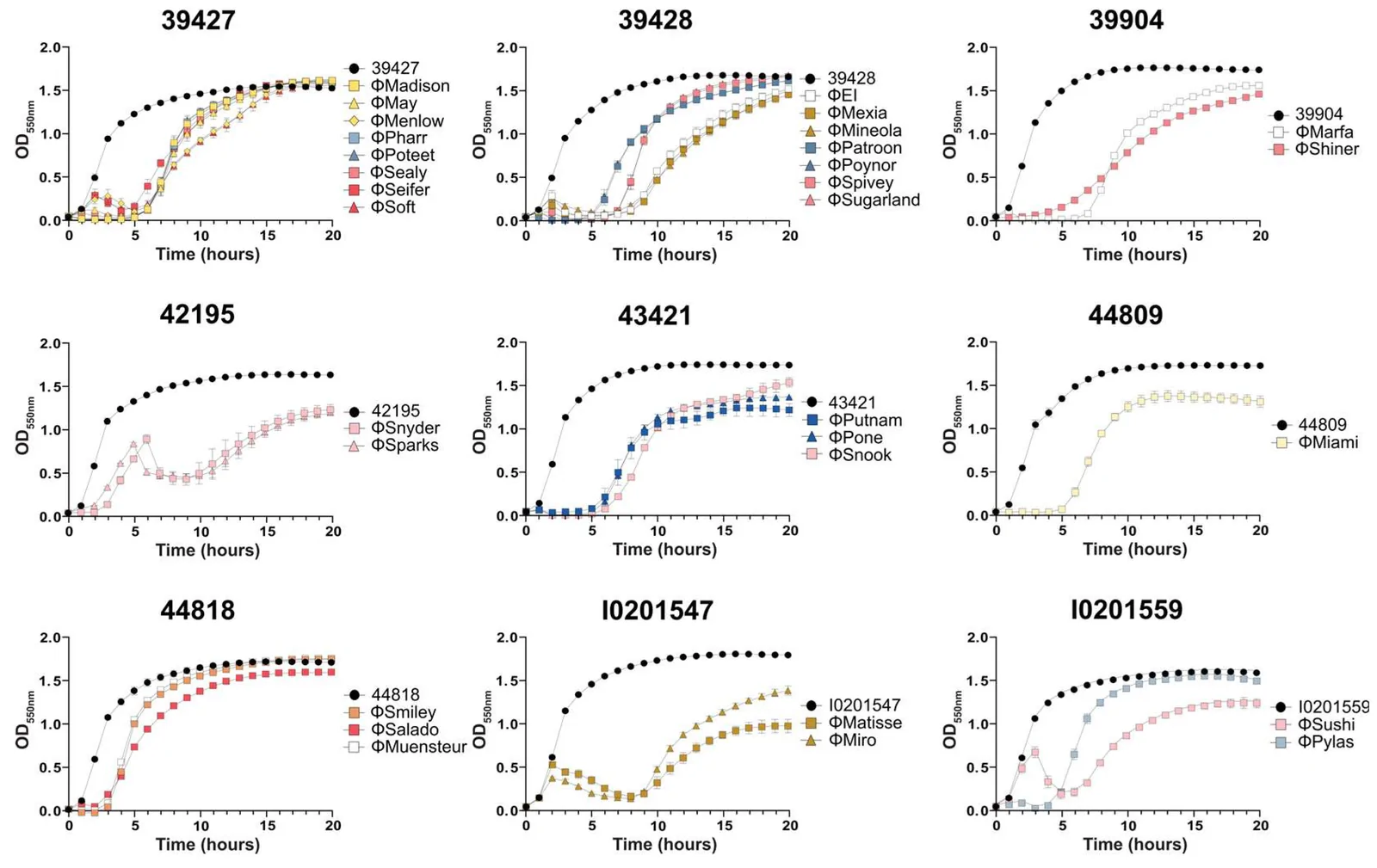
Lysis curves. A lysis curve of each bacteriophage was generated with its respective propagation host (Table 3) at an MOI of 0.1 in TSB. The lysis curve of each phage is colored based on taxonomic family previously used in Table 3. The black color is used for the propagation host without phage. The average of three independent biological replicates in triplicate is presented here.

**Figure 5:**
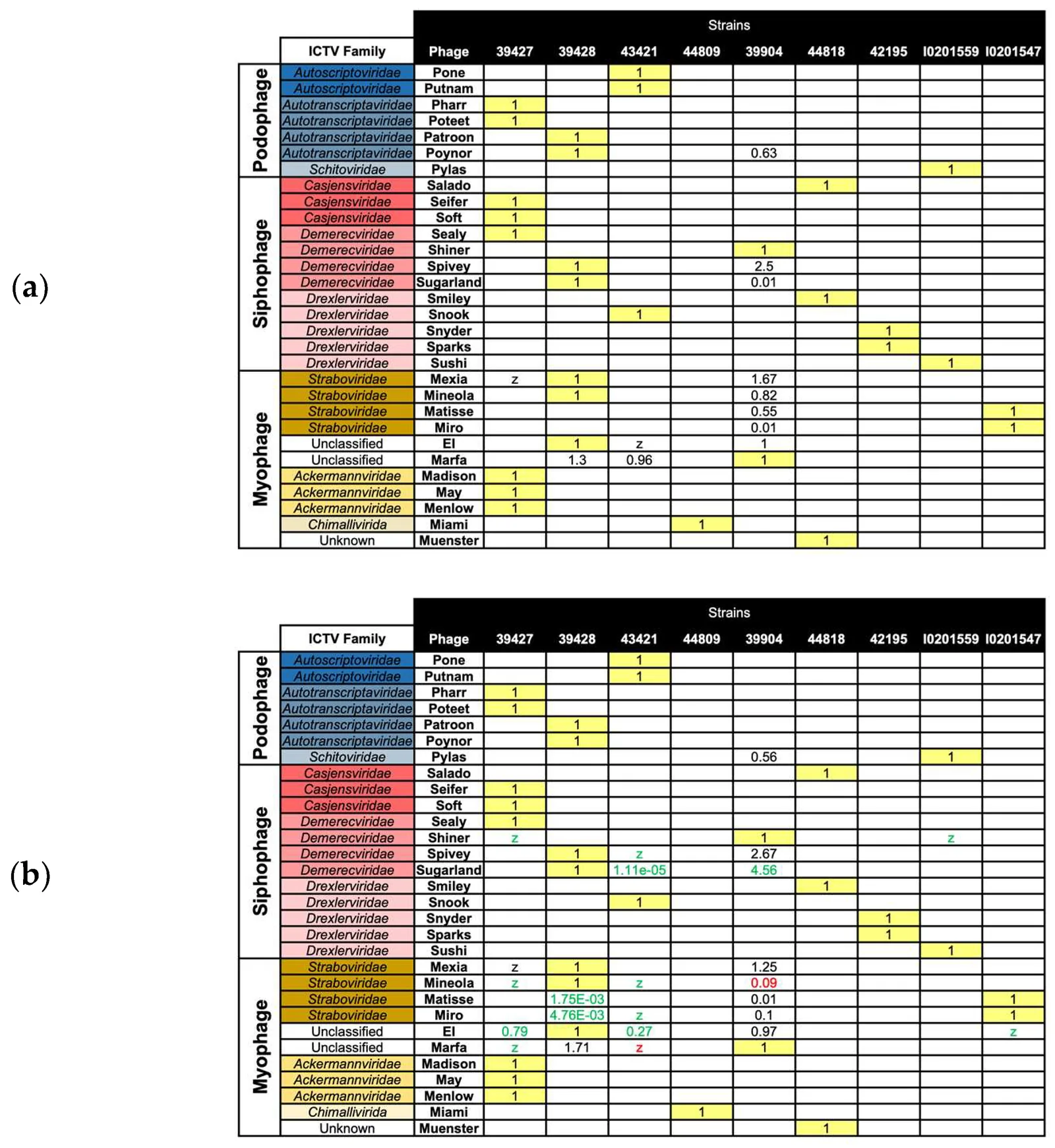
Effects of culture medium on phage sensitivity. Efficiency of plating (EOP) = 1 propagation host is denoted as yellow cells. A “Z” indicates the phage formed zones of clearing at high concentration but did not form plaques when further serially diluted. Blank cells indicate no interaction of the phage and strain at any concentration. Phages are separated into color groups based on their ICTV family designation using the same color scheme as used in Figure 3, and columns are labeled with the bacterial strains tested. (**a**) Host sensitivity to phages tested on TSB medium. (**b**) Host sensitivity to phages tested on LB medium. Differences in host range results compared to the TSB conduction are displayed in green for an increased sensitivity and red for decreased sensitivity to the same phages; only changes of tenfold or greater are highlighted. Sensitivities to most phages remain unchanged on LB; however, the sensitivity to phages in the *Straboviridae* (gold), *Demerecviridae* (red), and the unclassified myophages EI and Marfa were altered on LB compared to TSB. This indicates the choice of culture medium can affect apparent phage sensitivity in host range assays.

The host specificity and virulence of the 30 phages were determined using nine clinical *Klebsiella* spp. isolates (Figure 4 and Figure 5). A lysis curve of each phage with its respective propagation host in TSB at an MOI of 0.1 is shown in Figure 4. Half of the phages in the collection were propagated on 39427 or 39428, whereas strain 44809 has only a single phage propagated on it. The distribution of isolated phages is similar to that of *Klebsiella* spp. with common K loci, where K types 107 and 106 represent the first and third most prevalent K loci, respectively [51].

All phages demonstrated the ability to control bacterial growth in liquid culture, typically showing an initial reduction in culture followed by a rebound in bacterial growth after four to eight hours. Generally, the phages that were propagated on the same strain had similar lysis curves, even if they were unrelated. When examining the kinetics of bacterial growth in the presence of phage, growth curves could be divided into two main phenotypes: early inhibition of growth followed by rebound of the culture (e.g., phages Putnam or Pharr) or initial early bacterial growth followed by a reduction and then a rebound (e.g., phages Snyder or Sushi). The ability of the phages to control bacterial growth was quantified by dividing the area under the curves with phage treatment by the area under the control (no phage) curves (Supplementary Table S2). Based on this measurement, the *Straboviridae* phages Matisse, Mexia, Miro, Mineola, and EI had the strongest ability to control bacterial growth; this appears to be associated with a slightly more delayed and less severe growth rebound later in the experiment. Phages infecting isolate 44818 had the weakest lytic ability when quantified using this measure.

Plaque assays were conducted in both TSB and LB. Phages with the broadest host range in this panel on TSB, each capable of infecting three isolates, Mexia, Marfa, and EI, which belonged to the *Straboviridae* and unclassified families. Additionally, phages capable of infecting two isolates included Poynor in the *Autotranscriptaviridae* family; Spivey and Sugarland from the *Demerecviridae* family; and Mineola, Matisse, and Miro from the *Straboviridae* family. Most phages in the collection were only able to infect the isolate used for propagation. No interactions beyond the propagation phages were observed in strains 44809, 44818, 42195, I0201559, and I0201547.

The working culture system for phage experiments in this work was TSB medium; however, LB medium is also commonly used for propagation and characterization of *Klebsiella* phages. The infectivity of most phages was found to be consistent between the two media; however, the sensitivity of strains to phages in the *Straboviridae* and *Demerecviridae* families and the myophages EI and Marfa was found to be altered on LB compared to TSB. Phages Matisse and Miro were able to be plated with low efficiency on strain 39428 on LB. Phage Sugarland was able to plate with low efficiency on strain 43421, and phage EI displayed the ability to plate on strain 39427 and 43421 on LB.

### 3.4. Comparison of Predicted Phage Receptor-Binding Proteins

To better understand the proteins involved in phage receptor recognition, a set of 68 proteins with potential receptor-binding functions was extracted from the 30 phage genomes in this study (Table 4). These functions were determined based on the presence of conserved domains and homology to other known phage receptor-binding proteins based on BLASTp and HHpred searches. The phages in the collection were found to encode a wide variety of RBPs with diverse predicted arrangements, ranging from a single predicted RBP type in *Casjensviridae* and some *Autotranscriptaviridae* members, five to six predicted RBPs in the *Ackermannviridae*, and nine predicted RBPs in the jumbo phage Muenster (Table 4). A full list of proteins with accession numbers is provided in Supplementary Table S3. An analysis of phage RBPs for potential capsular depolymerase activity identified 39 proteins across 17 phages with depolymerase-associated conserved domains or similarity to known capsular depolymerases or sugar lyases as detected by InterProScan or HHpred (Supplementary Table S3, summarized in Table 4).

## 4. Discussion

The goal of this work was to characterize a diverse set of lytic phages that infect clinically relevant CR-KP isolates, which are capable of causing a range of difficult to treat infections due to their antibiotic resistance [3,7,79]. In this study, we determined phage genomic and growth characteristics to help identify phages that could be used for future clinical applications.

A portion of this work was performed to characterize the genomic features of the clinical isolates to gain better insight into the diversity of this collection. The nine carbapenem-resistant *Klebsiella* spp. isolates were selected as the minimally necessary strains to propagate the 30 phages reported here. The strains varied in sequence type (ST), with ST258 being the most represented (Table 2). ST258 is considered to be a major driving force for carbapenem resistance [8,12,14]. Additionally, ST17 and ST35 are common *K. pneumoniae* clonal lineages, yet not as overrepresented as ST258 [51]. While the remaining STs are less common than the previously mentioned types, inclusion of these within our study allows for a more comprehensive assessment of our phages against less common carbapenem-resistant *Klebsiella* spp. clinical isolates.

The capsule (K) locus diversity of the strain in this collection represents a wide variety of K types: KL22, KL25, KL51, KL102, KL106, KL107, KL125, and KL142. The capsule of *Klebsiella* can serve as both a physical barrier to prevent phage infection and as a phage receptor [24,29]. It is thus beneficial to have multiple K types within the collection since the capsule has been shown to be a major determinant of host tropism [24]. All strains in the collection possessed intact capsule loci, except strain 39428. This strain in the collection is predicted to be naturally acapsular due to the truncation of *wcaJ*, which has been shown to reduce capsule synthesis [80,81].

A proteomic tree was generated to display the genomic diversity of the phages presented in this work (Figure 3). The resulting tree provides support for the proposed classification of phages EI and Marfa within the *Marfavirus* family, while Muenster and phages of the *Alcyoneusvirus* genus are not clear members of any existing family. All phages displayed the ability to reduce bacterial growth in a liquid medium to varying degrees. Phages that were able to rapidly reduce bacterial growth in culture also selected for phage-insensitive populations that regrew in 6–12 h after inoculation. In some cases (e.g., phages Snyder or Sparks), there was an early period of bacterial growth followed by decline and later rebound; this may indicate phages with longer latent periods or smaller burst sizes that take longer to accumulate a large enough phage population to overwhelm the bacterial population. Phages propagated on the same host tended to exhibit similar phenotypes in this assay, indicating the bacterial host has an effect on phage virulence. However, it is difficult to disentangle here the properties of the phage from the host, as both can have an effect of phage activity. It is also not clear which phenotypes have any bearing on their therapeutic efficacy, but we note that phages Pharr, Soft, and Spivey have been shown to have therapeutic effects in rodent models [27,31].

While TSB and LB are common media for the culture of *K. pneumoniae*, this study used TSB as the standard culture medium [24,29]. Previous studies have observed the differences of achievable cell densities and phage titers comparing TSB and LB as a growth medium in *E. coli* [82], and these media can also produce differences in phage plating ability [83]. TSB differs in the nutrient content compared to LB, including the addition of dextrose; the presence of additional sugars in a medium has been shown to increase capsular polysaccharide production in multidrug-resistant *K. pneumoniae* [84]. We conducted simple host range assays using TSB and LB to determine if culture media affected the observed phage sensitivity. All phages with narrow host ranges on TSB maintained narrow host ranges on LB (Figure 5). However, the phages with broader host specificities on TSB showed an increase in host range when plated on LB. These changes in sensitivity may be attributed to production of capsule for several reasons. As noted above, *K. pneumoniae* strain 39428 is predicted to be acapsular, suggesting that phages able to plate on 39428 utilize a receptor other than capsule: Spivey, Sugarland, Mexia, Mineola, Matisse, Miro, EI, Marfa, Patroon, and Poynor [24,29,77,85]. These phages were able to infect strains of various K types when plated on both TSB and LB, indicating that the type of capsule produced is not a determinant for phage infection but rather that infection may be determined by the amount of capsule produced, which could mask the phage receptor if produced in sufficient quantity. Another phage that showed an expansion of host range on LB was Shiner, which was able to plate on strain 39427 on LB, yet not on TSB. Its propagation strain and 39427 have different K types, suggesting it may utilize a non-capsule receptor. The inability to plate on 39428 suggests potential intracellular factors or other surface features preventing successful infection. This observation highlights the need for rigorously standardized protocols to be able to compare phage sensitivity profiles between laboratories, as even “standard” media can produce variations in results.

Phages that recognize the bacterial capsule as their receptor often employ tail spikes with carbohydrate-degrading activity to adsorb to and digest their way through the capsule layer to reach the bacterial surface [85]. The presence of these depolymerase tail spikes across diverse phage families representing podo-, sipho- and myophage morphotypes demonstrates that the capsule may act as a potential receptor for many *Klebsiella* phages. Some phages appear to possess multiple depolymerase tail spikes, suggesting they may be able to infect cells expressing multiple capsule types. In the majority of cases (9/17 phages), only a single depolymerase tail spike was identified (Table 4). A common indicator for the presence of a phage-borne capsular depolymerase is the presence of a characteristic turbid halo surrounding the plaque proper when plated on soft agar lawns. While 15 phages produced haloed plaques suggesting capsular depolymerase activity (Figure 1, Table 4), the data shown here indicate this phenotype is not a reliable marker for phage receptor usage as some phages that have depolymerase tail spikes (Soft, Salado, Menlow, May, Madison) did not produce haloed plaques, while other phages that lack detectable depolymerases (Sushi, Patroon, Poynor) produced plaques with a halo phenotype. This indicates plaque morphology may not perfectly correlate with the presence of an identifiable depolymerase tail spike in the phage genome, as observed in previous studies [24,29,77,85]. Haloed plaque appearance is a qualitative measure for which the mechanism is not fully understood. Discrepancies between plaque observation and capsular depolymerase activity may be explained by variation in the amount or activity of excess enzyme produced during phage growth or the chemistry of the capsule [85]. Empirical determination of the phage receptors using genetic and/or biochemical methods is still required to confirm this specificity.

Previous studies on tail fiber arrangements in *Klebsiella* phages showed the presence of a wide variety of tail fiber structures in which multiple RBPs may be incorporated into a single complex branched tail fiber structure [86]. Phages in the collection were assigned predicted tail fiber arrangements based on their similarity to those described in a previous study (Table 4) [86]. In such cases, it is often not possible to determine which of these RBPs are responsible for recognition of a given host. The predicted RBPs were grouped by phage propagation host and compared using BLASTp to identify common sequences. Both phage Smiley and Muenster infect *K. pneumoniae* strain 44818, but these phages are otherwise unrelated. Specifically, Smiley is a small T1-like *Drexlerviridae* siphophage, and Muenster is an unclassified jumbo phage. However, the predicted Smiley depolymerase tail spike gp23 is clearly related to Muenster gp218, with 52% overall sequence identity that includes the predicted depolymerase domain. Given these two phages infect the same host, it is likely that of Muenster’s nine RBPs, gp218 is the protein responsible for host recognition. Likewise, the single depolymerase tail spike expressed by *Casjensviridae* siphophages Siefer and Soft shares 61% protein identity with *Ackermannviridae* phages May gp46, Menlow gp50, and Madison gp37. Although Madison, May, and Menlow encode six, five, and five RBPs, respectively, the fact that they share one RBP with Seifer/Soft that infects the same host suggests that these proteins are the ones used for host recognition in *K. pneumoniae* strain 39427. A single phage in the collection, Pharr, has an experimentally determined receptor, which is the capsule [87]. Pharr and Poteet tail spike proteins show high levels of amino acid identity, indicating that Poteet likely also uses the capsule as a receptor.

Four of the five *Drexlerviridae* phages in the collection appear to encode a central fiber and side tail spikes with carbohydrate-degrading activity. These five phages infect four distinct hosts, with only Snyder and Sparks infecting a common host. Across these phages, the predicted central tail fiber is highly conserved (86–94% sequence identity by BLASTp), while the side tail spikes are diverse. Only the Snyder and Sparks tail spikes are conserved (93% identity), while all other *Drexlerviridae* tail spikes are unrelated as indicated by BLASTp except for their extreme N-termini, which are the sites of virion attachment. These findings suggest that the side tail spikes are the major drivers of host specificity in these phages and that new specificity is commonly gained via horizontal gene transfer events to acquire novel receptor-binding domains rather than by genetic drift [86,88,89,90]. The lack of a detectable carbohydrate-degrading domain in the phage Sushi RBPs indicates this phage may recognize a non-capsule receptor for infection.

The findings presented here illustrate the limitations and potential for lytic phages against CR-KP. The variability in plating on different media shows the complexity in understanding phage–host interactions, as even changes in standard media can produce different results. This work also shows our limited understanding of non-capsular host receptor usage for *Klebsiella* phages. To enhance the potential utility of phages in the collection as therapeutics, it is necessary to improve our understanding of the receptors used by the phages in this collection.

## Figures and Tables

**Table 1:** Panel of *Klebsiella* spp. isolates used for phage propagation and characterization in this study. WCMC: Weill Cornell Medical College; NIH CC: National Institutes of Health Clinical Center.

Species	Strain	NCBI Accession	Source
*Klebsiella pneumoniae*	39427	NZ_NDBM00000000	M. Satlin, WCMC
*Klebsiella pneumoniae*	39428	NZ_NDBN00000000	M. Satlin, WCMC
*Klebsiella pneumoniae*	43421	NZ_NDDH00000000	M. Satlin, WCMC
*Klebsiella pneumoniae*	39904	NZ_NDBZ00000000	M. Satlin, WCMC
*Klebsiella quasipneumoniae*	I0201559	NA	K. Frank, NIH CC
*Klebsiella pneumoniae*	42195	NZ_NDEI00000000	M. Satlin, WCMC
*Klebsiella quasipneumoniae*	I0201547	NA	K. Frank, NIH CC
*Klebsiella pneumoniae*	44818	NZ_NDDT00000000	M. Satlin, WCMC
*Klebsiella pneumoniae*	44809	NZ_NDDK00000000	M. Satlin, WCMC

**Table 2:** Properties of *Klebsiella* spp. isolates used for phage propagation and characterization in this study. *bla*KPC allele type ND = not determined.

Species	Strain	Sequence Type	*bla*KPC Allele	Capsule (K) Locus Type	LPS O Antigen (O) Locus Type	No. of Phages Infecting Strain
*Klebsiella pneumoniae*	39427	258	KPC-3	107	O1/O2v2	9
*Klebsiella pneumoniae*	39428	258	KPC-2	106	O1/O2v2	8
*Klebsiella pneumoniae*	43421	17	KPC-2	25	O5	5
*Klebsiella pneumoniae*	39904	3585	KPC-2	111	O3b	2
*Klebsiella quasipneumoniae*	I0201559	3861	ND	142	O3/O3a	2
*Klebsiella pneumoniae*	42195	3586	Negative	102	O1/O2v1	2
*Klebsiella quasipneumoniae*	I0201547	359	ND	51	O1/O2v1	2
*Klebsiella pneumoniae*	44818	35	KPC-2	22	O1/O2v1	3
*Klebsiella pneumoniae*	44809	1502	KPC-3	125	O5	1

**Table 3:** Bacteriophages described in this study. Thirty bacteriophages infecting clinical *Klebsiella* spp. isolates were isolated from 2012 to 2019 from various sources, predominantly Texas municipal wastewater. Phage taxonomic classifications at the family and genus levels are provided based on the current ICTV taxonomy (2024 release). Morphology is predicted based on taxonomic classification. * Represents no current ICTV classification and use of BLASTn to determine closest relative in ICTV.

Phage Name	ICTV Family	ICTV Genus	NCBI Accession	Genome Size (bp)	Morphology	Isolation Source	Propagation Host	Citation
Pone	*Autoscriptoviridae*	*Drulisvirus*	MT701589	44,346	Podophage	Influent wastewater, Madison County, TX	43421	[62]
Putnam	Autoscriptoviridae *	Drulisvirus *	PQ358941	43,554	Podophage	Surface water, College Station, TX	43421	This work
Pharr	*Autotranscriptaviridae*	*Przondovirus*	MK618658	40,599	Podophage	Influent wastewater, College Station, TX	39427	[27]
Poteet	Autotranscriptaviridae *	Przondovirus *	PQ299796	41,093	Podophage	Influent wastewater, Madison County, TX	39427	This work
Patroon	*Autotranscriptaviridae*	*Teetrevirus*	MK608335	39,442	Podophage	Influent wastewater, Bryan, TX	39428	[63]
Poynor	Autotranscriptaviridae *	Teetrevirus *	PQ299797	39,792	Podophage	Influent wastewater, College Station, TX	39428	This work
Pylas	*Schitoviridae*	*Pylasvirus*	MH899585	70,408	Podophage	Influent wastewater, College Station, TX	I0201559	[64]
Salado	Casjensviridae *	Yonseivirus *	PQ358942	57,380	Siphophage	Surface water, College Station, TX	44818	This work
Seifer	*Casjensviridae*	*Yonseivirus*	MH817999	58,197	Siphophage	Influent wastewater, Brazos County, TX	39427	[65]
Soft	*Casjensviridae*	*Yonseivirus*	MN106244	57,805	Siphophage	Influent wastewater, Houston, TX	39427	[27]
Sealy	Demerecviridae *	Sugarlandvirus *	PQ337358	113,355	Siphophage	Influent wastewater, College Station, TX	39427	This work
Shiner	Demerecviridae *	Sugarlandvirus *	PQ283208	113,813	Siphophage	Activated wastewater sludge, College Station, TX	39904	This work
Spivey	*Demerecviridae*	*Sugarlandvirus*	MK630230	118,049	Siphophage	Influent wastewater, College Station, TX	39428	[27]
Sugarland	*Demerecviridae*	Sugarlandvirus *	NC_042093	111,103	Siphophage	Influent wastewater, College Station, TX	39428	[66]
Smiley	Drexlerviridae *	Webervirus *	PQ337355	49,197	Siphophage	Influent wastewater, Houston, TX	44818	This work
Snook	Drexlerviridae *	Webervirus *	PQ337356	49,393	Siphophage	Influent wastewater, Houston, TX	43421	This work
Snyder	Drexlerviridae *	Webervirus *	PQ337357	49,722	Siphophage	Influent wastewater, College Station, TX	42195	This work
Sparks	Drexlerviridae *	Webervirus *	PQ358943	49,937	Siphophage	Influent wastewater, College Station, TX	42195	This work
Sushi	*Drexlerviridae*	*Webervirus*	KT001920	48,754	Siphophage	Influent wastewater, College Station, TX	I0201559	[67]
Mexia	Straboviridae *	Jiaodavirus *	PQ368522	166,124	Myophage	Influent wastewater, Buffalo, NY	39428	This work
Mineola	*Straboviridae*	*Jiaodavirus*	MH333064	166,130	Myophage	Activated wastewater sludge, Bryan, TX	39428	[68]
Matisse	*Straboviridae*	*Slopekvirus*	NC_028750	176,081	Myophage	Influent wastewater, College Station, TX	I0201547	[69]
Miro	Straboviridae *	Slopekvirus *	KT001919	176,055	Myophage	Influent wastewater, College Station, TX	I0201547	[70]
EI	Unclassified	Marfavirus *	MN106245	170,423	Myophage	Influent wastewater, Kansas City, KS	39428	This work
Marfa	Unclassified	*Marfavirus*	MN044033	168,532	Myophage	Pooled swine fecal samples, Texas and Michigan	39904	[71]
Madison	Ackermannviridae *	Taipeivirus *	PQ368523	157,834	Myophage	Influent wastewater, Austin, TX	39427	This work
May	*Ackermannviridae*	*Taipeivirus*	MG428991	159,631	Myophage	Influent wastewater, Bryan, TX	39427	[72]
Menlow	*Ackermannviridae*	*Taipeivirus*	MG428990	157,281	Myophage	Influent wastewater, College Station, TX	39427	[73]
Miami	*Chimallivirida*	*Miamivirus*	MT701590	253,383	Myophage	Influent wastewater, College Station, TX	44809	[74]
Muenster	Unclassified	*Alcyoneusvirus*	MT708547	346,937	Myophage	Influent wastewater, College Station, TX	44818	[75]

**Table 4:** Annotated receptor-binding proteins (RBPs) in 30 *K. pneumoniae* phage genomes. Proteins were included based on conserved domains or similarity to other annotated phage proteins based on BLASTp or HHpred searches, and 70 proteins from phages in the collection were used in the study. Phages were found to encode from one to nine potential RBPs, with 17 of the 30 phages encoding at least one RBP with predicted carbohydrate-degrading activity. Here, the term tail spike refers to a receptor-binding protein with predicted carbohydrate-degrading enzymatic activity, whereas tail fibers are predicted to act as adhesins without enzymatic activity.

ICTV Family	Phage	Halo Plaque	Host	No. Predicted RBPs	No. RBPs with Predicted Carbohydrate-Degrading Activity	Predicted RBP Arrangement
*Ackermannviridae*	Madison	-	39427	6	4	Branched multi-fiber arrangement
*Ackermannviridae*	May	-	39427	5	4	Branched multi-fiber arrangement
*Ackermannviridae*	Menlow	-	39427	5	4	Branched multi-fiber arrangement
*Autoscriptoviridae*	Pone	Y	43421	2	2	Dual branched tail spikes
*Autoscriptoviridae*	Putnam	Y	43421	1	1	Side tail spikes
*Autotranscriptaviridae*	Patroon	Y	39428	1	0	Side tail fiber adhesins
*Autotranscriptaviridae*	Poynor	Y	39428	1	0	Side tail fiber adhesins
*Autotranscriptaviridae*	Pharr	Y	39427	2	2	Dual branched tail spikes
*Autotranscriptaviridae*	Poteet	Y	39427	2	2	Dual branched tail spikes
*Casjensviridae*	Salado	-	44818	1	1	Side tail spikes
*Casjensviridae*	Seifer	Y	39427	1	1	Side tail spikes
*Casjensviridae*	Soft	-	39427	1	1	Side tail spikes
*Chimallivirida*	Miami	Y	44809	6	5	Branched multi-fiber arrangement
*Demerecviridae*	Sealy	-	39427	2	0	Side adhesin tail fibers, central fiber with tip adhesin
*Demerecviridae*	Shiner	-	39904	2	0	Side adhesin tail fibers, central fiber with tip adhesin
*Demerecviridae*	Spivey	-	39428	2	0	Side adhesin tail fibers, central fiber with tip adhesin
*Demerecviridae*	Sugarland	-	39428	2	0	Side adhesin tail fibers, central fiber with tip adhesin
*Drexlerviridae*	Smiley	Y	44818	2	1	Side tail spikes, central adhesin fiber
*Drexlerviridae*	Snook	Y	43421	2	1	Side tail spikes, central adhesin fiber
*Drexlerviridae*	Snyder	Y	42195	2	1	Side tail spikes, central adhesin fiber
*Drexlerviridae*	Sparks	Y	42195	2	1	Side tail spikes, central adhesin fiber
*Drexlerviridae*	Sushi	Y	I0201559	2	0	Side adhesin tail fibers, central adhesin fiber
*Schitoviridae*	Pylas	Y	I0201559	1	1	Side tail spikes
*Straboviridae*	Matisse	-	I0201547	1	0	T4-like long tail fibers, terminal adhesin
*Straboviridae*	Miro	-	I0201547	1	0	T4-like long tail fibers, terminal adhesin
*Straboviridae*	Mexia	-	39428	1	0	T4-like long adhesin tail fibers
*Straboviridae*	Mineola	-	39428	1	0	T4-like long adhesin tail fibers
Unclassified	EI	-	39428	1	0	T4-like long adhesin tail fibers
Unclassified	Marfa	-	39904	1	0	T4-like long adhesin tail fibers
Unclassified	Muenster	Y	44818	9	7	Branched multi-fiber arrangement

## Data Availability

Phage genome and protein sequences are available from NCBI under the accession numbers provided in the manuscript.

## Supplementary Materials

The following supporting information can be downloaded at: https://www.mdpi.com/article/10.3390/v18101079/s1, Table S1: Minimum inhibitory concentrations (MICs) and breakpoints for clinical bloodstream *K. pneumoniae* isolates collected from New York and New Jersey medical centers in 2013. Table S2: Numerical quantification of phage virulence assays (Figure 4). Table S3: Phage characteristics and predicted properties of phage receptor-binding proteins.

## Abbreviations

The following abbreviations are used in this manuscript:

CR-KP	Carbapenem-resistant *K. pneumoniae*
ESBL	Extended spectrum beta-lactamase
LB	Luria broth
LA	Luria agar
TSB	Tryptic soy broth
TSA	Tryptic soy agar
K	Capsule
O	O antigen
MLST	Multilocus sequence typing
MOI	Multiplicity of infection
ICTV	International Committee on Taxonomy of Viruses
PFU	Plaque-forming units
CFU	Colony-forming units
